# Mental health in Germany in the first weeks of the Russo-Ukrainian war

**DOI:** 10.1192/bjo.2023.21

**Published:** 2023-04-14

**Authors:** Cornelia Gottschick, Sophie Diexer, Janka Massag, Bianca Klee, Anja Broda, Oliver Purschke, Mascha Binder, Daniel Sedding, Thomas Frese, Matthias Girndt, Jessica I. Hoell, Patrick Michl, Michael Gekle, Rafael Mikolajczyk

**Affiliations:** Institute for Medical Epidemiology, Biometry and Informatics, Interdisciplinary Centre for Health Sciences, Medical Faculty of the Martin-Luther-University Halle-Wittenberg, Germany; Department of Internal Medicine IV – Oncology/Haematology, Martin-Luther-University Halle-Wittenberg, Germany; Mid-German Heart Centre, Department of Cardiology and Intensive Care Medicine, University Hospital, Martin-Luther-University Halle-Wittenberg, Germany; Institute of General Practice and Family Medicine, Interdisciplinary Centre for Health Sciences, Medical Faculty of the Martin-Luther-University Halle-Wittenberg, Germany; Department of Internal Medicine II, Martin-Luther-University Halle-Wittenberg, Germany; Paediatric Haematology and Oncology, Martin-Luther-University Halle-Wittenberg, Germany; Department of Internal Medicine I, Martin-Luther-University Halle-Wittenberg, Germany; Julius Bernstein-Institute of Physiology, Faculty of Medicine, Martin-Luther-University Halle-Wittenberg, Germany

**Keywords:** Anxiety disorders, depressive disorders, epidemiology, rating scales, statistical methodology

## Abstract

**Background:**

In the connected world, although societies are not directly involved in a military conflict, they are exposed to media reports of violence.

**Aims:**

We assessed the effects of such exposures on mental health in Germany during the military conflict in Ukraine.

**Method:**

We used the German population-based cohort for digital health research, DigiHero, launching a survey on the eighth day of the Russo-Ukrainian war. Of the 27 509 cohort participants from the general population, 19 444 (70.7%) responded within 17 days. We measured mental health and fear of the impact of war compared with other fears (natural disasters or health-related).

**Results:**

In a subsample of 4441 participants assessed twice, anxiety in the population (measured by the Generalised Anxiety Disorder-7 screener) was higher in the first weeks of war than during the strongest COVID-19 restrictions. Anxiety was elevated across the whole age spectrum, and the mean was above the cut-off for mild anxiety. Over 95% of participants expressed various degrees of fear of the impact of war, whereas the percentage for other investigated fears was 0.47–0.82. A one-point difference in the fear of the impact of war was associated with a 2.5 point (95% CI 2.42–2.58) increase in anxiety (11.9% of the maximum anxiety score). For emotional distress, the increase was 0.67 points (0.66–0.68) (16.75% of the maximum score).

**Conclusions:**

The population in Germany reacted to the Russo-Ukrainian war with substantial distress, exceeding reactions during the strongest restrictions in the COVID-19 pandemic. Fear of the impact of war was associated with worse mental health.

After 2 years of the COVID-19 pandemic, with its accompanied varying degrees of restriction and emotional distress, the world's population was confronted with the Russo-Ukrainian war.^1^ Military conflicts strongly affect those directly involved.^2^ For example, post-traumatic stress disorder (PTSD) is a health condition that can be triggered by war events and is commonly found in soldiers and veterans.^3^ Furthermore, the civilian population suffers from psychological distress after traumatic events or as a consequence of displacement, resulting in an increase in the number of mental disorders and psychosomatic complaints.^4–6^ Women, children, the elderly and the disabled are most affected by war events.^7^ However, distress can also result from exposure to graphic media images, and affect persons not directly involved in the military conflict.^8,9^ Although economic effects in neighbouring countries of war zones have been analysed,^10^ it is not known how military conflicts affect mental health in countries of relatively close geographic and political proximity. Therefore, we aimed to quantify the mental health effects in the German population during the first weeks of the Russo-Ukrainian war.

## Method

We used the sample of the population-based prospective cohort study for digital health research in Germany (DigiHero, German Clinical Trials Register (DRKS) identifier: DRKS00025600). We initiated DigiHero in the city of Halle (Saxony-Anhalt, Germany) during the COVID-19 pandemic, and invited participants through regular mail. The study was subsequently extended to the federal state of Saxony-Anhalt and other selected federal states in Germany (Saxony and Bavaria). Up to the beginning of March 2022, 27 509 individuals had agreed to participate in DigiHero. Except for the paper-based invitation (to establish population-based recruitment), participation in the study is digital. Registration includes a baseline questionnaire with sociodemographic variables and opening questions on health-related aspects. The study includes further questionnaires and potential invitations to additional study components.

We started the current survey on 4 March 2022, the eighth day after the Russian army's invasion of Ukraine, and closed the questionnaire on 21 March 2022, after two reminders. We applied standardised instruments to assess mental health and reduced the reference period to 1 week. Anxiety was measured with the Generalised Anxiety Disorder-7 (GAD-7) screener. The GAD-7 is a seven-item scale measuring symptom severity on a scale from ‘Not at all’ (0) to ‘Nearly every day’ (3), with a total severity score ranging from 0 to 21, with higher scores indicating higher anxiety. Depressive symptoms were measured with the Patient Health Questionnaire-9 (PHQ-9), using the same 0–3 scale as the GAD-7 but for nine items, for a total score ranging from 0 to 27. Well-being was assessed with the World Health Ogranization-5 Well-Being Index comprising five items, on a scale from ‘At no time’ (0) to ‘All of the time’ (5), for a total score of 0–25, with a higher score indicating better well-being. Stress was measured with the Perceived Stress Questionnaire (PSQ-20), which has 20 items and uses a scale from ‘Almost never’ (0) to ‘Usually’ (3), for a total score ranging from 0 to 60. During the COVID-19 pandemic, we assessed the mental health of the participants in the then available subsample of the DigiHero cohort (*n* = 7796). This substudy was conducted in September 2021 with the GAD-7 and PHQ-9. The participants were asked questions in relation to the current situation (which was a time of moderate COVID-19 pandemic-related restrictions), and about March 2021 (the period of strongest COVID-19 pandemic-related restrictions in Germany). A total of 4441 participants provided responses for both time periods. These data were available for comparison with mental health assessed in March 2022. In addition, to measure fear of the impact of war, we used a scale allowing a comparative assessment of potential individually frightening events, modifying an instrument used by the German National Cohort (Supplementary Table 1 available at https://doi.org/10.1192/bjo.2023.21).^11^ Finally, by rephrasing items and the introductory question, we adapted the Peritraumatic Distress Inventory (PDI) to the situation of ongoing exposure to the occurring war (for details, see Supplementary Appendix 1 and Supplementary Table 2).^12^

### Statistical analysis

We report data as means and s.d. or as frequencies. For the analysis of the modified PDI scale, we used an exploratory factor analysis with varimax rotation and the minimum residual solution for the number of subscales.^13^ We derived two subscales: ‘emotional distress’ (Cronbach's α = 0.848) and ‘physical distress’ (Cronbach's α = 0.789), calculated as mean value of the respective items (for details, see Supplementary Tables 6–11 and Supplementary Figs 1 and 2). The proportions of participants with GAD-7 scores representing mild (score ≥5), moderate (score ≥10) and severe (score ≥15) levels of anxiety symptoms were determined.^14^ In the next step, we graphically compared the levels of anxiety and depressive symptoms during the periods of the pandemic and the beginning of war, by gender and age. To study the relationship between anxiety, depressive symptoms, emotional and physical distress and age in detail (i.e. allowing for non-linearity), we employed generalised additive models as implemented in the R library *mgcv* (with splines for age, identity link, and normal distribution for the dependent variable).^15^ We also used generalised additive models to study the associations between fear of war and mental health outcomes adjusting for age, gender, income, being in a partnership, federal state and living in a city with >100 000 inhabitants. Education and country of birth were excluded from the multivariable analysis, as they showed no association in the unadjusted model. Point estimates and their 95% confidence intervals were obtained. All models were checked for normality of the residuals. To compare the strength of the associations between mental health and fear of the impact of war, we expressed the difference per one unit of the fear scale as a percentage of the maximum score for each studied mental health instrument. Because item-level incompleteness in the mental health measures was extremely low, we performed a complete-case analysis. All analyses were conducted with R version 4.1.0 for Windows (R Core Team, Vienna, Austria; https://www.R-project.org/).^15,16^

### Ethics

The authors assert that all procedures contributing to this work comply with the ethical standards of the relevant national and institutional committees on human experimentation and with the Helsinki Declaration of 1975, as revised in 2008. All procedures involving human patients were approved by the Ethics Committee of the Martin-Luther University Halle-Wittenberg (approval number 2020-076) (DigiHero, German Clinical Trials Register (DRKS) identifier: DRKS00025600). Written informed consent was obtained from all participants, using the online study portal with individual registrations.

## Results

Of the 27 509 invited DigiHero participants, 19 444 (70.7%) completed the survey (Supplementary Table 3). Participants who did not respond displayed similar characteristics to those who did, although there were more missing values for sociodemographic data in the group of non-responders (Supplementary Table 4).

**Fig. 1:**
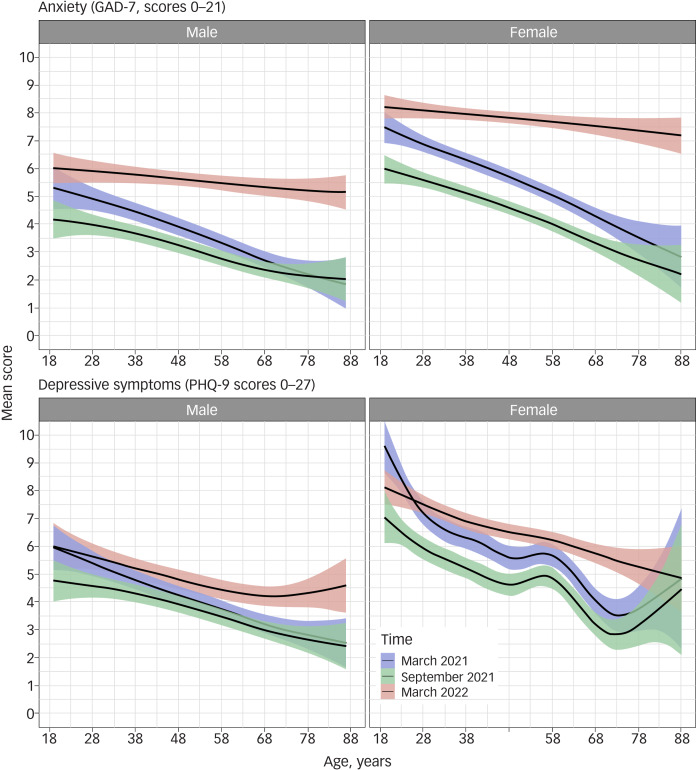
Mean scores of anxiety (GAD-7) and depressive symptoms (PHQ-9) at two time points during the pandemic (with stronger restrictions in March 2021 and moderate restrictions in September 2021) and in March 2022 (immediately after the Russian invasion of Ukraine), stratified by gender (a subsample of 4441 participants from the city of Halle). Numbers for the whole sample of participants who answered the questionnaire inquiring about the war in Ukraine were only available for March 2022, but these were virtually the same as the estimates in the subsample for Halle. Figures are obtained by ggplot, applying the *gam* function, with splines in the *mgcv* library. GAD-7, Generalised Anxiety Disorder-7; PHQ-9, Patient Health Questionnaire-9.

Mental health (anxiety and depressive symptoms) was worse in the first weeks of war compared with the period of strongest restrictions during the COVID-19 pandemic in Germany (Fig. 1). Although the mental health of older age groups was less affected during the pandemic than the mental health of younger age groups, this difference was less pronounced during the early days of the Russo-Ukrainian war, particularly in terms of anxiety. Median anxiety at the population level (as measured by the GAD-7 score) was 6 (interquartile range (IQR): 3–10) in March 2022 compared with 3 (IQR: 0–6) in September 2021 and 4 (IQR: 1–7) in March 2021.

In March 2022, 9.73% had GAD-7 scores corresponding to severe anxiety levels; this percentage was 5.02% in March 2021 and 3.20% in September 2021. Conversely, in the period of the beginning of the war in March 2022, 35.5% of participants had a GAD-7 score representing minimal anxiety symptoms; this percentage was 63.1 in September 2021 and 52.7 in March 2021 (Table 1).

**Table 1:** Percentage of participants with minimal, mild, moderate and severe levels of anxiety severity, measured by the Generalised Anxiety Disorder-7, over time

Generalised Anxiety Disorder-7 anxiety severity^a^	March 2021, %	September 2021, %	March 2022, %
Minimal (0–4)	52.65	63.14	35.51
Mild (5–9)	29.88	25.04	35.60
Moderate (10–14)	09.81	07.59	17.32
Severe (15–21)	05.02	03.20	09.73

a. Based on reference values by Spitzer et al.^14^

**Fig. 2:**
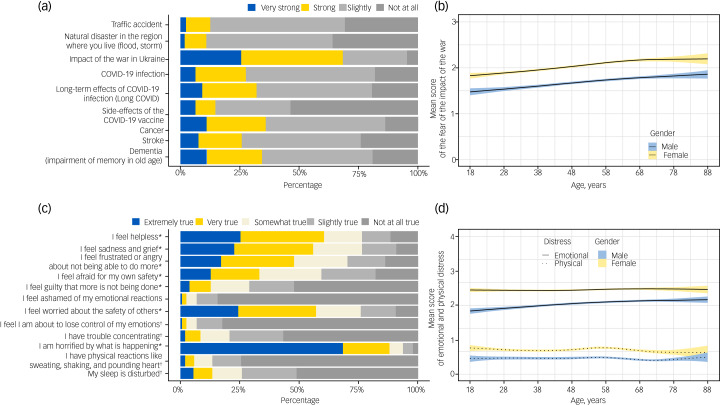
(a) Answers to the question ‘Please indicate whether you, for yourself, are afraid of the following events’. (b) Mean score of fear of the impact of the war in Ukraine (from 0 ‘not at all’ to 3 ‘very strong’) by gender and age. (c) Answers to the question ‘Which statements apply to you regarding the Ukraine crisis?’ (modified Peritraumatic Distress Inventory). *Items summarised to ‘emotional distress’; ^†^items summarised to ‘physical distress’. (d) Mean score of emotional and physical distress subscales related to the war in Ukraine (from 0 ‘Not at all true’ to 4 ‘Extremely true’). Lines in (b) and (d) were obtained using ggplot with the *mgcv* library in R, with splines for age, identity link and normal distribution for the outcome variables.

Fear of the impact of war in Ukraine was rated higher than other fears enquired about, including those related to the COVID-19 pandemic (Fig. 2(a)). Over 95% of the participants expressed various degrees of fear of the impact of war, whereas the percentage for other investigated fears was 0.47%–0.82%.

**Fig. 3:**
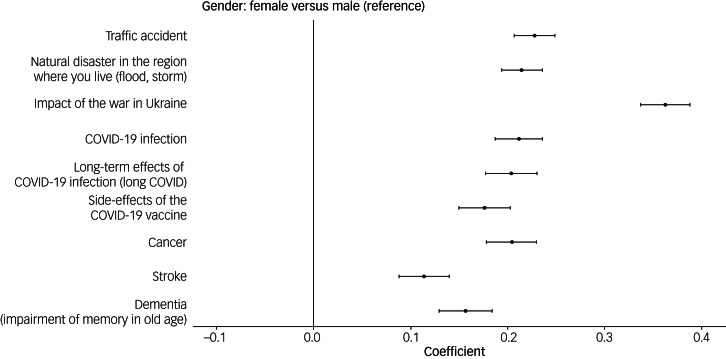
Association of gender with different fears, adjusted for age, federal state, living in a big city, household income and being in a partnership. Point estimates and 95% confidence intervals are shown (coefficients indicate differences on the outcome scale, age is modelled nonlinearly).

**Fig. 4:**
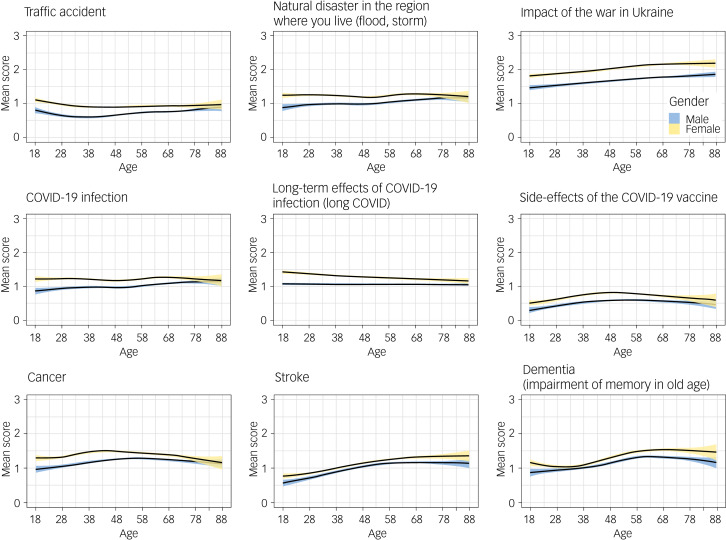
Mean score of studied fears (scores 0–3) by gender and age. Lines are obtained from ggplot employing the *mgcv* library in R, and indicate non-linear effects of age, modelled as splines, with normal distribution for outcome variable, and identity link.

Women scored their fear of the impact of war 0.37 points higher than men, and there was a nearly linear increase by age in both gender (0.04 points per 10 years; Fig. 2(b)). In addition, living in a partnership resulted in higher fear scores (Supplementary Table 5). Similar effects were observed across all levels of education (data not shown). Being born in Germany and having their own child living in the same household did not have any effect. The differences in fear scores by gender were less pronounced for fears unrelated to the Russo-Ukrainian war (Fig. 3). There was lower or no correlation with age for other fears (Fig. 4).

With respect to items measuring peritraumatic distress, those indicating emotional distress were scored substantially higher than items related to physical reactions (Fig. 2(c)). The highest score was achieved by the item: ‘I am horrified by what is happening’. There were almost no differences by age in the subscales denoting emotional and physical distress, but women had a higher mean score for both emotional and physical distress than men (Fig. 2(d)).

Higher fear of the impact of war was associated with higher anxiety and depressive symptoms, worse well-being, higher stress, and higher emotional and physical distress (Table 2). An increase of one point for ‘fear of the impact of war (0–4)’, for example, was associated with an increase of 2.5 points in anxiety score (GAD-7, range 0–21). The association was stronger for anxiety, emotional distress and physical distress, and less pronounced for depressive symptoms and stress. There was an interaction between fear and gender with respect to mental health outcomes, with stronger associations in women than men.

**Table 2:** Association of mental health with fear of the impact of war (generalised additive models)

Mental health scales	Crude model *ß* (95% CI)	Adjusted model *ß* (95% CI)^a^	Percentage of maximum score^b^
Anxiety (GAD-7, scores 0–21)	2.6 (2.52–2.67)	2.5 (2.42–2.58)	11.9
Depressive symptoms (PHQ-9, scores 0–27)	1.43 (1.35–1.52)	1.42 (1.33–1.5)	5.26
Well-being (WHO-5, scores 0–25)	−1.77 (−1.87 to −1.68)	−1.71 (−1.8 to −1.61)	6.84
Stress (PSQ-20, scores 0–60)	4.02 (3.8–4.23)	4.1 (3.89–4.31)	6.83
Emotional distress (mPDI, scores 0–4)	0.69 (0.67–0.7)	0.67 (0.66–0.68)	16.75
Physical distress (mPDI, scores 0–4)	0.36 (0.34–0.37)	0.35 (0.34–0.36)	8.75

Numbers indicate differences in mental health scores between groups differing by one point of fear score; the numbers correspond to coefficients in linear regression. GAD-7, Generalised Anxiety Disorder-7; PHQ-9, Patient Health Questionnaire-9, Depression Module; WHO-5, World Health Organization-5 Well-Being Index; PSQ-20, Perceived Stress Questionnaire; mPDI, modified Peritraumatic Distress Inventory.

a. For age, gender, federal state, living in a big city, household income and in a partnership.

b. Adjusted difference expressed as percentage of the maximum score of the corresponding instrument.

## Discussion

In the early weeks of the Russo-Ukrainian war, mental health in the German population was worse than during the strongest restrictions related to the COVID-19 pandemic. Anxiety was strongly elevated, with the average score for the whole study population exceeding the cut-off for mild anxiety symptom levels.^14,17,18^ Fear of the impact of war far exceeded fears of individual adverse events or health-related events. Those with stronger fear also had worse mental health, particularly with respect to anxiety and emotional distress as measured by the modified PDI.

In 2008, a study on anxiety in the German general population showed that approximately 5% of participants had a moderate level of anxiety and 1% had a severe level of anxiety.^18^ In comparison, our study showed a substantially higher percentage of participants with high anxiety levels in all survey periods. In March 2021, this was likely linked to the ongoing COVID-19 pandemic and to strong pandemic-related restrictions. Although in September 2021, in the period of moderate pandemic restrictions, mental health was better overall, the anxiety score was still high compared with 2008. In March 2022, some pandemic restrictions still existed and possibly influenced mental health. The Russo-Ukrainian war thus hit the German population during the ongoing pandemic and likely increased anxiety levels. Nevertheless, the anxiety levels in March 2022 displayed different characteristics to the earlier (pandemic) time points, with much higher levels in older age groups. During the COVID-19 pandemic, several studies observed that older people were less distressed than younger age groups.^1,19^ Fear of COVID-19 infection targets the individual perspective, and there were reports that older people considered the risk of death ‘natural’. The pattern of higher levels of fear among younger age groups compared with older age groups observed during the pandemic did not persist in March 2022. In the current study, fear of the impact of war increased with age; emotional distress was age-dependent in men and increased with age. It appears that older age groups are especially affected. War is not a natural event, and its consequences can very broadly affect the life of whole societies. Therefore, future studies are needed to understand how its various dimensions may contribute to the overall deterioration of mental health during a time of war.

Women had more often symptoms of anxiety, depression, and emotional and physical distress than men. Higher levels of negative health outcomes among women are well known, and there are some reports of stronger reactions to traumatising events among women.^20–22^ A study from Israel analysed factors associated with mental health during the COVID-19 pandemic and a period of political tension.^23^ Eshel et al analysed the interaction between various aspects of mental health, but did not directly report differences in health for the two periods.^24^ In addition, the involvement of the Israeli study population in the conflict was much more direct than the situation analysed in our study.

The mechanisms by which the war in Ukraine can affect mental health in Germany include media reports of violence as well as medial presence of the war in political and societal life, which can generate concerns regarding one's own future. In the early days of the war, these fears were based on anticipation of negative effects rather than experience, because neither shortages nor high prices had started. Asking broadly about the consequences of the war in the first days, we did not assess exactly which fears respondents had. Furthermore, the extent of the war's presence in the media might not truly reflect the objective severity of the war, but be intertwined with aspects like geographic, political and cultural proximity.

We found that a much larger proportion of participants were affected by fear of the impact of the war in Ukraine at the time of the survey than by other fears related to COVID-19 infection, or to health risks such as stroke. Based on these data, we cannot answer whether and how long this effect and the symptoms will persist, or if adaption to the stressor will occur. It is also unclear whether indirect exposure to war affects mental health after the end of the military conflict. A recent review indicated that various consequences may follow exposure to stressful conditions including epidemics and natural disasters, such as worsening of pre-existing disorders, develop of acute stress disorder (PTSD) or development of subclinical symptomatic stress disorder.^25^ This could suggest that the burden of the war in countries not involved might not be negligible.

Because of media coverage, modern society has achieved a condition in which these military conflicts are present permanently, even in societies not directly affected. Although they occur all over the world, the Russo-Ukrainian war entered a new dimension of international involvement. The high level of emotional distress observed in our study may be derived from feelings of uncertainty, empathy, frustration or sadness, as well as from fears for one's own safety, thereby resulting in mixed dimensions of sympathy and own involvement.^26,27^

### Strengths and limitations

A major strength of this study is the large sample with full coverage of regions in which the study was conducted. The ability to study mental health in almost real time was facilitated by the digital research approach. The sample was recruited independently of the current topic. Any selection was probably skewed toward a positive attitude to participation in health studies, but not to mental health or war. Within the study sample, we achieved a high and very timely response, and we did not observe strong selection effects with respect to the measured characteristics among those who responded and those who did not. There was only a slightly lower response among younger people, people with a migration background or people with a lower income. This is in line with a generally lower participation among groups with these characteristics in studies. At the time of this study, the sample from DigiHero was restricted to three federal states. In terms of standard indicators, these federal states are among the most different within Germany. Yet, our results indicated only minor regional differences regarding the studied outcomes, after adjusting for individual sociodemographic characteristics. We cannot claim representativeness for the whole of Germany, but consider the lack of regional differences in our analysis as an indication that regional differences might not be very important. Nevertheless, we note some limitations. By its very nature, DigiHero is restricted to participants with internet access. In a previous work, we demonstrated that among individuals willing to participate in a research study, responses were virtually the same when paper-based participation was also allowed.^28^ Compared with the general population, our sample was more educated and had a higher income. Nevertheless, in our sample we did not observe differences in fear of the impact of war according to education or income. Regarding the high levels of anxiety and depressive symptoms, it could be that mental health in the beginning of the war was also still affected by the consequences of the COVID-19 pandemic, and at an individual level, by symptoms caused by the infection. Although we assume that exposure to the topic of war in the media facilitates the observed reactions, we did not measure media exposure in our survey. Additionally, we do not have any information on previous psychiatric diagnoses, which may likely affect instruments used in this study, thus leaving this question open for future research. Another limitation is that were not able to identify standardised instruments for the measurement of fear of war. Consequently, we had to develop a scale comparing different fears. Similarly, to our knowledge, a validated instrument measuring trauma related to prolonged exposure to war in another country did not exist. We therefore had to modify the existing PDI instrument for use in our study. On the one hand, we cannot refer to established validity of the original instrument. On the other hand, we consider that the measured items have high face validity and might therefore be fit for the purpose.

Overall, military conflicts affect mental health beyond the immediate areas of destruction. The German population responded strongly to the war in Ukraine, with fear of the impact of war, anxiety and distress. The effects on mental health during the initial weeks of the conflict exceeded those of the strongest restrictions related to the COVID-19 pandemic. Overall, we think that there is a need to study the mental health effects of war in countries not directly involved.

## Data Availability

The anonymised data reported in this study can be obtained from the corresponding author, R.M., upon request. The data-set includes individual data, and an additional data dictionary will be provided. The beginning of data availability starts with the date of publication, and the authors will support any requests in the three following years. Data requests should include a proposal for the planned analyses. Decisions will be made according to data use by the access committee of the DigiHero study, and data transfer will require a signed data access agreement.

## Supplementary material

For supplementary material accompanying this paper visit http://doi.org/10.1192/bjo.2023.21. click here to view supplementary material
