# Supplementary material for: Mental health in Germany in the first weeks of the Russo-Ukrainian war
Source: BJPsych Open. 2023 Apr 14;9(3):e66. doi: 10.1192/bjo.2023.21 (PMC10134205; doi:10.1192/bjo.2023.21)
Supplement: Supplementary file 1 [file S2056472423000212sup001.docx]

# **SUPPLEMENTARY MATERIALS**

**Table of Contents**

[Supplementary method 2](#_Toc98783818)

[Supplementary Tables 3](#_Toc98783819)

[Supplementary Figures 8](#_Toc98783820)

[References 11](#_Toc98783821)

## **Supplementary method**

**Modification of the Peritraumatic Distress Inventory**

The PDI was initially developed to allow assessment of the PTSD criteria.^1^ It focusses on describing extreme reactions at the time of the traumatic event. In such way, it was not meant for the long-term exposure to distressing events or conditions. Unfortunately, our search of an appropriate instrument for distress caused by war reported by media was unsuccessful. We therefore decided to modify PDI for the needs of the current study. The introductory question of the original PDI was modified to “Which statements apply to you regarding the Ukraine crisis?” In the next step, past tense in the items related to the events were replaced by present tense, reflecting the continuous exposure. Furthermore, the item related to loss of control of bowel and bladder was replaced by the item “I have trouble concentrating”. Finally, the items “I felt I might pass out” and “I thought I might die” were replaced by the item “my sleep is disturbed”. The response options were maintained (Table S2).

**Construction of subscales**

Exploratory factor analysis with Varimax rotation was applied to study internal structure of the modified PDI. The original PDI employs two subscales, and the wording of items suggested an emotional and physical factor. We applied the minimum residual (minres) solution to assess the number of factors.^2^ The two factors that were retained in the analysis accounted for 44.98% of the total variance of the original data (MR1 = 25·47%, MR2 = 19·51%, Table S5). Item loadings on the two factors are shown in Figure S3.

Subsequently, internal reliability of the two scales was investigated. We compared overall Cronbach’s Alpha for each factor to the Alpha values after a particular item was deleted (Table S6, Table S7). The deletion of the items “guilty” (“I feel guilty that not more is being done”) and “ashamed” (“I feel ashamed of my emotional reactions”) led to an increase in Alpha values (0·848 and 0·788 respectively). Hence, these items were removed from the first and second factor, respectively (Table S8, Table S9).

## **Supplementary Tables**

**Table S1:** Scale used for measurement of fears. (Adaption from NAKO Questionnaire)

| **Questions** | **Answers** |
| --- | --- |
| Please indicate whether you, for yourself, are afraid of the following events.   1. Traffic accident 2. Natural disaster in the region where you live (flood, storm) 3. Impact of the war in Ukraine 4. Coronavirus infection 5. Long-term effects of the Coronavirus infection (LongCOVID) 6. Side effects of the corona vaccine 7. Cancer 8. Stroke 9. Dementia (Impairment of memory in old age) | Very strong  Strong  Slightly  Not at all |

**Table S2:** The modified Peritraumatic Distress Inventory (Adaption of the Peritraumatic Distress Inventory (PDI))

| **Questions** | **Answers** |
| --- | --- |
| Which statements apply to you regarding the Ukraine crisis?   1. I feel helpless. 2. I feel sadness and grief. 3. I feel frustrated and angry about not being able to do more. 4. I fear for my own safety. 5. I feel guilty that more is not being done. 6. I am ashamed of my emotional reactions. 7. I am worried about the safety of others. 8. I feel I am about to lose control of my emotions 9. I have trouble concentrating. 10. I am horrified by what is happening. 11. I have physical reactions like sweating, shaking, and my heart pounding 12. My sleep is disturbed. | Not at all True  Slightly True  Somewhat True  Very True  Extremely True |

**Table S3.** Socio-demographic characteristics of study participants (N (%))

|  |  | **Male** | **Female** | **Total*** |
| --- | --- | --- | --- | --- |
|  | | 7,722 (39·7) | 11,009 (56·6) | 19,444 (100·0) |
| Age | |  |  |  |
|  | 18–29 | 612 (7·9) | 1359 (12·3) | 1992 (10·2) |
|  | 30–39 | 1107 (14·3) | 2011 (18·3) | 3133 (16·1) |
|  | 40–49 | 1150 (14·9) | 1974 (17·9) | 3138 (16·1) |
|  | 50–59 | 1621 (21·0) | 2716 (24·7) | 4361 (22·4) |
|  | 60–69 | 1838 (23·8) | 1996 (18·1) | 3861 (19·9) |
|  | 70+ | 1300 (16·8) | 844 (7·7) | 2159 (11·1) |
|  | *Missing* | 94 (1·2) | 109 (1·0) | 800 (4·1) |
| Born in Germany | |  |  |  |
|  | Yes | 7453 (96·5) | 10713 (97·3) | 1992 (10·2) |
|  | No | 248 (3·2) | 279 (2·5) | 3133 (16·1) |
|  | Not specified/Unknown | 16 (0·2) | 8 (0·1) | 3138 (16·1) |
|  | *Missing* | 5 (0·1) | 9 (0·1) | 4361 (22·4) |
| Education level | |  |  |  |
|  | Low | 239 (3·1) | 575 (5·2) | 826 (4·2) |
|  | Medium | 2155 (27·9) | 3422 (31·1) | 5616 (28·9) |
|  | High | 4989 (64·6) | 6550 (59·5) | 11610 (59·7) |
|  | *Missing* | 339 (4·4) | 462 (4·2) | 1392 (7·2) |
| Household income** | |  |  |  |
|  | <1750 | 858 (11·1) | 1612 (14·6) | 2500 (12·9) |
|  | 1750–3000 | 2219 (28·7) | 3032 (27·5) | 5278 (27·1) |
|  | 3000–4000 | 1647 (21·3) | 2203 (20·0) | 3875 (19·9) |
|  | 4000–5000 | 1150 (14·9) | 1649 (15·0) | 2811 (14·5) |
|  | >5000 | 1356 (17·6) | 1543 (14·0) | 2921 (15·0) |
|  | Not specified/Unknown | 474 (6·1) | 943 (8·6) | 1428 (7·3) |
|  | *Missing* | 18 (0·2) | 27 (0·2) | 631 (3·2) |
| In partnership | |  |  |  |
|  | Yes | 6292 (81·5) | 8524 (77·4) | 14906 (76·7) |
|  | No | 1304 (16·9) | 2309 (21·0) | 3646 (18·8) |
|  | Not specified/Unknown | 90 (1·2) | 145 (1·3) | 237 (1·2) |
|  | *Missing* | 36 (0·5) | 31 (0·3) | 655 (3·4) |
| Own child (<18 years) living in same household | |  |  |  |
|  | Yes | 1643 (21·3) | 2943 (26·7) | 4608 (23·7) |
|  | No | 6016 (77·9) | 8026 (72·9) | 14155 (72·8) |
|  | *Missing* | 63 (0·8) | 40 (0·4) | 681 (3·5) |
| Federal state | |  |  |  |
|  | Saxony-Anhalt | 5217 (67·6) | 7652 (69·5) | 13357 (68·7) |
|  | Saxony | 1191 (15·4) | 1593 (14·5) | 2860 (14·7) |
|  | Bavaria | 1199 (15·5) | 1616 (14·7) | 2925 (15·0) |
|  | Other | 84 (1·1) | 140 (1·3) | 263 (1·4) |
|  | *Missing* | 31 (0·4) | 8 (0·1) | 39 (0·2) |
| Living in a big city (>100,000 inhabitants) | |  |  |  |
|  | Yes | 3142 (40·7) | 4924 (44·7) | 8345 (42·9) |
|  | No | 4482 (58·0) | 5971 (54·2) | 10853 (55·8) |
|  | *Missing* | 98 (1·3) | 114 (1·0) | 246 (1·3) |
| * 20 (0·1%) participants reported diverse sex | |  |  |  |
| ** Average net monthly household income in Germany is 3,612 euros | | | | |

**Table S4:** Socio-demographic characteristics of responders vs. non-responders.

|  |  | **Responder** | **Non-Responder** | **Total** |
| --- | --- | --- | --- | --- |
| N (%) | | 19444 (70·7) | 8065 (29·3) | 27509 (100·0) |
| Age | |  |  |  |
|  | 18-29 | 1992 (10·2) | 1397 (17·3) | 3389 (12·3) |
|  | 30-39 | 3133 (16·1) | 1418 (17·6) | 4551 (16·5) |
|  | 40-49 | 3138 (16·1) | 1129 (14·0) | 4267 (15·5) |
|  | 50-59 | 4361 (22·4) | 1222 (15·2) | 5583 (20·3) |
|  | 60-69 | 3861 (19·9) | 919 (11·4) | 4780 (17·4) |
|  | 70+ | 2159 (11·1) | 593 (7·4) | 2752 (10·0) |
|  | *missing* | 800 (4·1) | 1387 (17·2) | 2187 (8·0) |
| Sex | |  |  |  |
|  | Male | 7722 (39·7) | 3256 (40·4) | 10978 (39·9) |
|  | Female | 11009 (56·6) | 3460 (42·9) | 14469 (52·6) |
|  | Diverse | 20 (0·1) | 16 (0·2) | 36 (0·1) |
|  | *Missing* | 693 (3·6) | 1333 (16·5) | 2026 (7·4) |
| Born in Germany | |  |  |  |
|  | Yes | 18287 (94·0) | 6413 (79·5) | 24700 (89·8) |
|  | No | 529 (2·7) | 331 (4·1) | 860 (3·1) |
|  | Not specified/Unknown | 26 (0·1) | 24 (0·3) | 50 (0·2) |
|  | *Missing* | 602 (3·1) | 1297 (16·1) | 1899 (6·9) |
| Education level | |  |  |  |
|  | Low | 826 (4·2) | 588 (7·3) | 1414 (5·1) |
|  | Medium | 5616 (28·9) | 2140 (26·5) | 7756 (28·2) |
|  | High | 11610 (59·7) | 3667 (45·5) | 15277 (55·5) |
|  | *Missing* | 1392 (7·2) | 1670 (20·7) | 3062 (11·1) |
| Household income | |  |  |  |
|  | <1750 | 2500 (12·9) | 1301 (16·1) | 3801 (13·8) |
|  | 1750 - 3000 | 5278 (27·1) | 1791 (22·2) | 7069 (25·7) |
|  | 3000 - 4000 | 3875 (19·9) | 1139 (14·1) | 5014 (18·2) |
|  | 4000 - 5000 | 2811 (14·5) | 802 (9·9) | 3613 (13·1) |
|  | >5000 | 2921 (15·0) | 1030 (12·8) | 3951 (14·4) |
|  | Not specified/Unknown | 1428 (7·3) | 656 (8·1) | 2084 (7·6) |
|  | *Missing* | 631 (3·2) | 1346 (16·7) | 1977 (7·2) |
| In partnership | |  |  |  |
|  | Yes | 14906 (76·7) | 5125 (63·5) | 20031 (72·8) |
|  | No | 3646 (18·8) | 1487 (18·4) | 5133 (18·7) |
|  | Not specified/Unknown | 237 (1·2) | 134 (1·7) | 371 (1·3) |
|  | *Missing* | 655 (3·4) | 1319 (16·4) | 1974 (7·2) |
| Own child (<18 years) living in same household | |  |  |  |
|  | Yes | 14906 (76·7) | 5125 (63·5) | 6429 (23·4) |
|  | No | 3646 (18·8) | 1487 (18·4) | 19068 (69·3) |
|  | *Missing* | 237 (1·2) | 134 (1·7) | 2012 (7·3) |
| Federal state | |  |  |  |
|  | Saxony-Anhalt | 13357 (68·7) | 5931 (73·5) | 19288 (70·1) |
|  | Saxony | 2860 (14·7) | 688 (8·5) | 3548 (12·9) |
|  | Bavaria | 2925 (15·0) | 1168 (14·5) | 4093 (14·9) |
|  | Other | 263 (1·4) | 216 (2·7) | 479 (1·7) |
|  | *Missing* | 39 (0·2) | 62 (0·8) | 101 (0·4) |
| Living in a big city (>100.000 inhabitants) | |  |  |  |
|  | Yes | 8345 (42·9) | 4005 (49·7) | 12350 (44·9) |
|  | No | 10853 (55·8) | 3804 (47·2) | 14657 (53·3) |
|  | *Missing* | 246 (1·3) | 256 (3·2) | 502 (1·8) |
|  | |  |  |  |

**Table S5:** Association of Fear of the impact of the war in Ukraine with sociodemographic (multivariable generalized additive model, GAM as implemented in mgcv library in R, normal distribution and identity link).

| Outcome: Fear of the impact of the war in Ukraine | | | |
| --- | --- | --- | --- |
| R^2^ = 0·059 N = 18177 | | B (95% CI) | *p* |
| Age | | Smooth Curve, EDF = 6·861 | <0·001 |
| Sex | |  |  |
|  | Male | Ref |  |
|  | Female | 0·37 (0·34;0·39) | <0·001 |
|  | Diverse | 0·12 (-0·27; 0·52) | 0·537 |
| Income | |  |  |
|  | >5000 | Ref |  |
|  | 4000 - 5000 | 0·04 (-0·01; 0·09) | 0·002 |
|  | 3000 - 4000 | 0·10 (0·06; 0·14) | 0·008 |
|  | 1750 - 3000 | 0·05 (0·01; 0·10) | <0·001 |
|  | <1750 | 0·07 (0·02; 0·11) | 0·079 |
|  | Not specified/unknown | 0·10 (0·05; 0·16) | <0·001 |
| In partnership | |  |  |
|  | Yes | Ref |  |
|  | No | -0·12 (-0·15; -0·08) | <0·001 |
|  | Not specified/unknown | -0·18 (-0·29; -0·07) | 0·001 |
| Federal state | |  |  |
|  | Saxony-Anhalt | Ref |  |
|  | Saxony | -0·05 (-0·08; -0·01) | 0·008 |
|  | Bavaria | -0·05 (-0·09; -0·02) | 0·002 |
|  | Other | 0·14 (-0·1; 0·37) | 0·244 |
| Living in a big city | |  |  |
|  | Yes | Ref |  |
|  | No | 0·06 (0·03; 0·08) | <0·001 |

Ref- reference category

**Table S6:** Summary of the explorative factor analysis.

| Factor | Eigenvalue | Explained Variance | Variance_Cumulative | Variance_Proportion |
| --- | --- | --- | --- | --- |
| MR1 | 4·043030 | 0·2547225 | 0·2547225 | 0·5663175 |
| MR2 | 1·354414 | 0·1950649 | 0·4497875 | 0·4336825 |

### **Table S7:** Item analysis on Factor 1.

|  | Mean | SD | Skew | Item Difficulty | Item Discrimination | Alpha, if deleted |
| --- | --- | --- | --- | --- | --- | --- |
| helpless | 2·52 | 1·29 | -0·63 | 0·70 | 0·641 | 0·815 |
| sadness | 3·47 | 1·24 | -0·5 | 0·69 | 0·727 | 0·802 |
| frustrated | 2·21 | 1·29 | -0·31 | 0·64 | 0·710 | 0·804 |
| fearforsafety_own | 1·87 | 1·28 | 0·09 | 0·57 | 0·569 | 0·827 |
| guilty | 0·94 | 1·18 | 1·02 | 0·39 | 0·416 | 0·848 |
| fearforsafety_other | 2·49 | 1·26 | -0·52 | 0·70 | 0·611 | 0·820 |
| shocked | 3·49 | 0·92 | -2·06 | 0·90 | 0·533 | 0·833 |

|  |  |
| --- | --- |
|  | Overall Cronbach’s Alpha=0·844 |

###

### **Table S8:** Item analysis on Factor 2.

|  | Mean | SD | Skew | Item Difficulty | Item Discrimination | Alpha, if deleted |
| --- | --- | --- | --- | --- | --- | --- |
| ashamed | 0·26 | 0·69 | 3·1 | 0·25 | 0·322 | 0·788 |
| loosecontroloffeelings | 0·27 | 0·68 | 2·9 | 0·25 | 0·599 | 0·722 |
| problems_with_concentration | 0·74 | 1·03 | 1·33 | 0·35 | 0·643 | 0·689 |
| physical_reactions | 0·46 | 0·93 | 2·14 | 0·29 | 0·619 | 0·700 |
| sleep_disordered | 0·94 | 1·2 | 1·15 | 0·39 | 0·593 | 0·719 |

|  |  |
| --- | --- |
|  | Overall Cronbach’s Alpha=0·770 |

**Table S9:** Item analysis on Factor 1 after item deletion “guilty”.

|  | Mean | SD | Skew | Item Difficulty | Item Discrimination | Alpha, if deleted |
| --- | --- | --- | --- | --- | --- | --- |
| helpless | 2·52 | 1·29 | -0·63 | 0·70 | 0·654 | 0·818 |
| sadness | 2·47 | 1·24 | -0·5 | 0·69 | 0·735 | 0·802 |
| frustrated | 2·21 | 1·29 | -0·31 | 0·64 | 0·692 | 0·811 |
| fearforsafety_own | 1·87 | 1·28 | 0·09 | 0·57 | 0·569 | 0·835 |
| fearforsafety_other | 2·49 | 1·26 | -0·52 | 0·70 | 0·604 | 0·828 |
| shocked | 3·49 | 0·92 | -2·06 | 0·90 | 0·546 | 0·839 |

|  |  |
| --- | --- |
|  | Overall Cronbach’s Alpha=0·848 |

**Table S10:** Item analysis on Factor 2 after item deletion “ashamed”.

|  | Mean | SD | Skew | Item Difficulty | Item Discrimination | Alpha, if deleted |
| --- | --- | --- | --- | --- | --- | --- |
| loosecontroloffeelings | 0·27 | 0·68 | 2·9 | 0·25 | 0·567 | 0·766 |
| problems_with_concentration | 0·74 | 1·03 | 1·33 | 0·35 | 0·644 | 0·711 |
| physical_reactions | 0·46 | 0·93 | 2·14 | 0·29 | 0·630 | 0·721 |
| sleep_disordered | 0·94 | 1·2 | 1·15 | 0·39 | 0·616 | 0·741 |

|  |  |
| --- | --- |
|  | Overall Cronbach’s Alpha=0·788 |

**Table S11:** Summary of the factor analysis on the final items.

| \| Component \| Eigenvalue \| Explained Variance \| Variance_Cumulative \| Variance_Proportion \| \| --- \| --- \| --- \| --- \| --- \| \| MR1 \| 3·73 \| 0·29 \| 0·29 \| 0·57 \| \| MR2 \| 1·28 \| 0·22 \| 0·50 \| 0·43 \| |  |  |  |  |
| --- | --- | --- | --- | --- | --- | --- | --- | --- | --- | --- | --- | --- | --- | --- | --- | --- | --- | --- | --- |
|  |  |  |  |  |
|  |  |  |  |  |

## **Supplementary Figures**


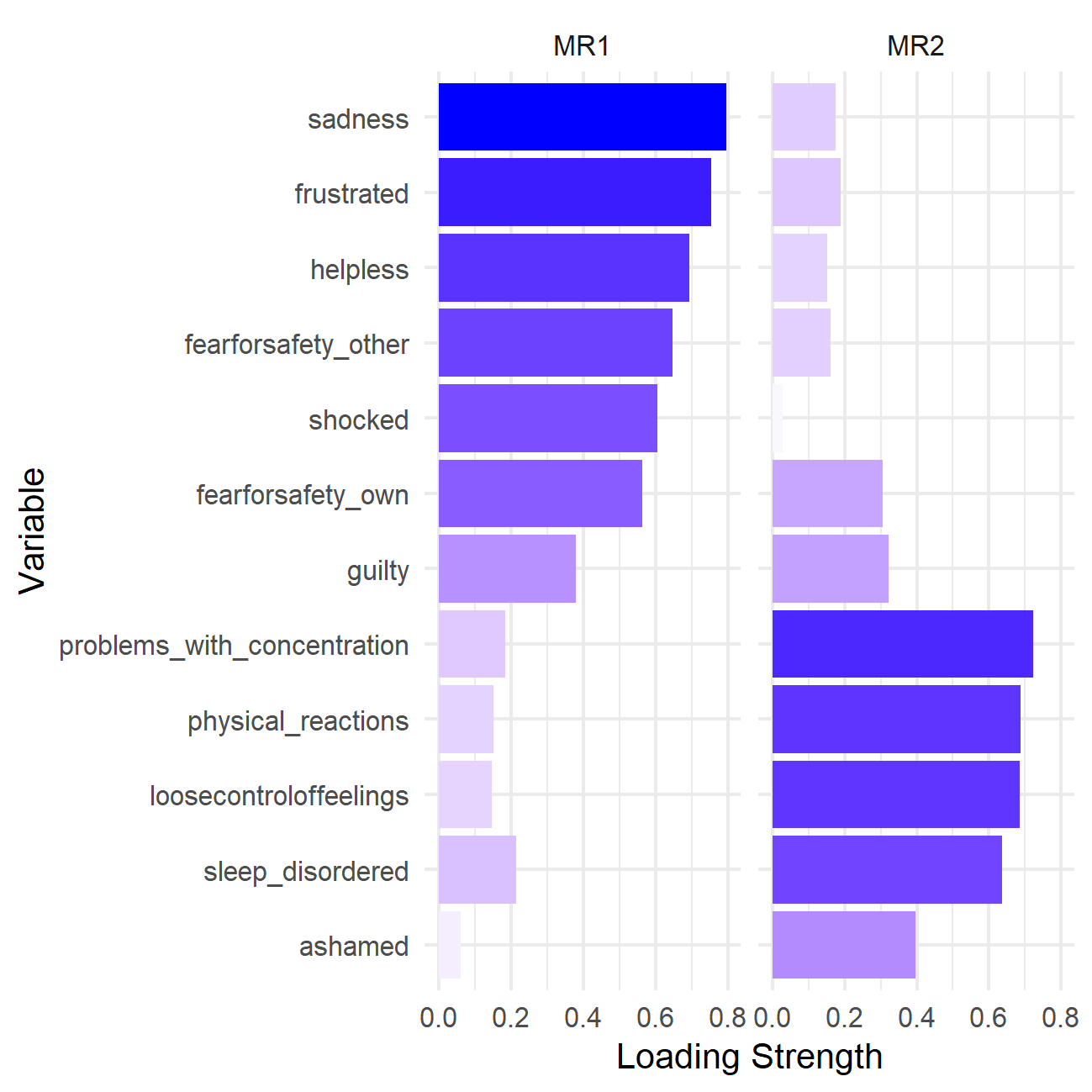


**Figure S1.** Item loadings on the two factors (MR1, MR2).


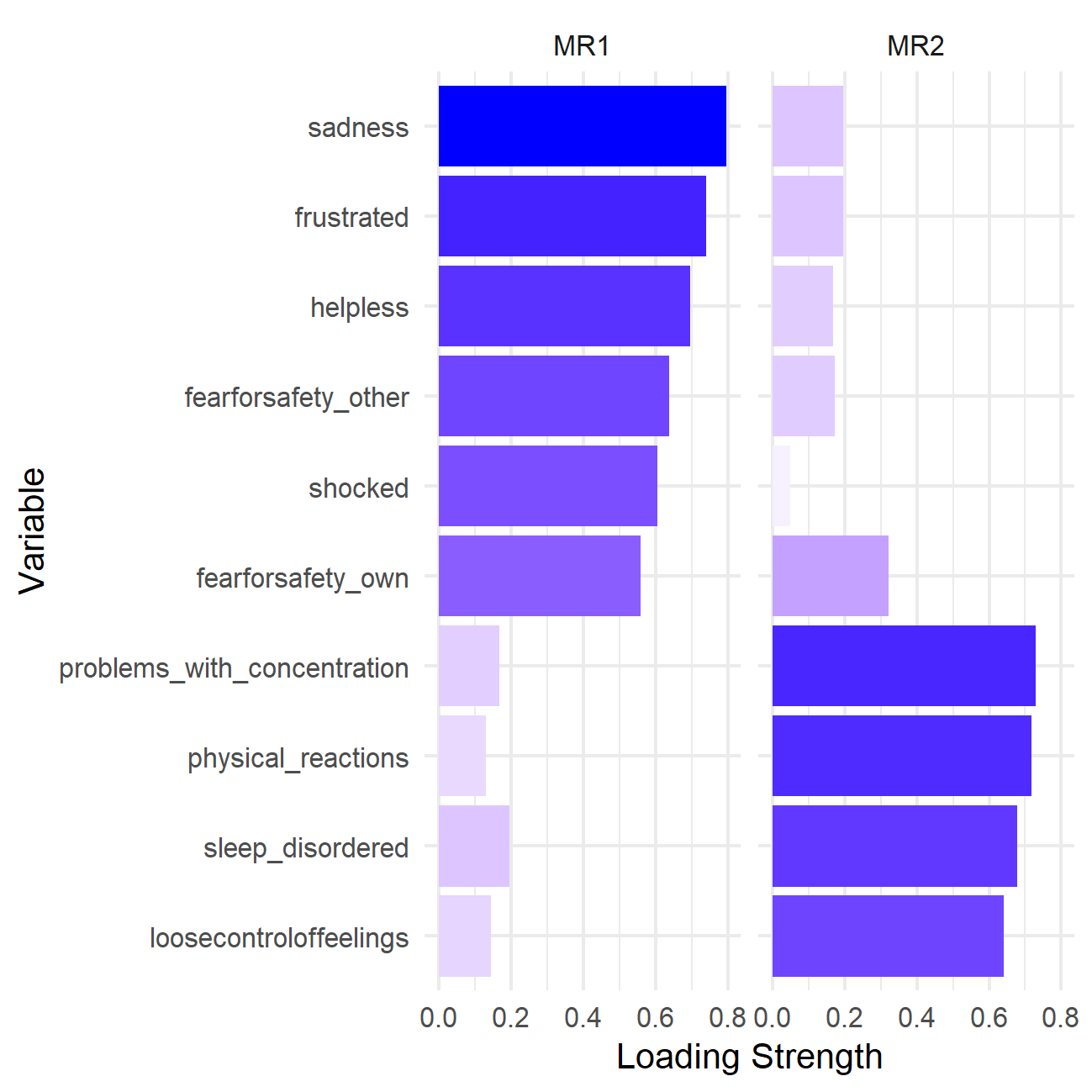


**Figure S2.** Loadings of the final items on the two factors (MR1, MR2).

## **References**

1. Brunet, A. *et al.* The Peritraumatic Distress Inventory: A Proposed Measure of PTSD Criterion A2. *Am. J. Psychiatry* **158**, 1480–1485 (2001).

2. Harman, H. H. & Jones, W. H. Factor analysis by minimizing residuals (minres). *Psychometrika* **31**, 351–368 (1966).
